# Experiences of Home‐Based Pulmonary Rehabilitation With mHealth and Centre‐Based Pulmonary Rehabilitation in People With Chronic Obstructive Pulmonary Disease: A Qualitative Study

**DOI:** 10.1111/hex.70480

**Published:** 2025-10-30

**Authors:** Wen Sun, Caixia Qiu, Shungui Xu

**Affiliations:** ^1^ The Affiliated People's Hospital of Fujian University of Traditional Chinese Medicine Fuzhou Fujian Province China

I have recently read the article titled ‘Experiences of Home‐Based Pulmonary Rehabilitation With mHealth and Centre‐Based Pulmonary Rehabilitation in People With Chronic Obstructive Pulmonary Disease: A Qualitative Study’ published in HEALTH EXPECTATIONS. I am writing this letter to commend Hannah Rutherford and her colleagues for this groundbreaking research.

Poor adherence to pulmonary rehabilitation exercises among patients with Chronic Obstructive Pulmonary Disease (COPD) has long been a major challenge in clinical monitoring [1, 2]. Actively exploring convenient and humanised monitoring approaches to improve patients' compliance with pulmonary rehabilitation is of crucial importance in today's ageing society.

This study proposes a highly feasible mobile health application (mHealth) service model and conducts an in‐depth exploration of patients' experiences with a mHealth application‐supported pulmonary rehabilitation (m‐PR) and centre‐based pulmonary rehabilitation (CB‐PR). By analysing multiple dimensions of information, including patients' personal preferences, social environments and available resources, the study finds that under mHealth management, personalised exercise scenarios and convenient doctor–patient communication can enhance patients' willingness to participate [3].

While this study offers valuable insights, it has certain limitations. First, the sample size is obviously small, and the average age of participants is relatively high, and most are retired, which may prevent it from fully representing the real situation of COPD patients in the general population. Meanwhile, the participants in this study generally have a relatively high level of literacy. Consequently, the research findings may not be applicable to populations from other cultural backgrounds or those with low digital literacy, which is unfavourable for the popularisation of mHealth solutions. Second, the observation period is only 8 weeks, which is mismatched with the long‐term intervention nature of pulmonary rehabilitation. This short timeframe fails to truly reflect the actual impacts of the two pulmonary rehabilitation models on patients' long‐term pulmonary function indicators, quality of life and the sustainability of exercise habits. Finally, the study did not assess participants' lifestyle factors, such as nutritional intake and daily exercise, which may interfere with the research results and affect the accurate judgement of differences in effectiveness and patient experience between the two pulmonary rehabilitation models.

To enhance the persuasiveness of the study, I put forward the following suggestions:

1. Expand the sample size and balance the sample structure: conduct stratified sampling based on dimensions such as age, occupation type and educational background. Meanwhile, take targeted measures (e.g., optimising the mHealth system) to improve accessibility for populations with low digital literacy—this not only helps expand the sample size but also reduces the impact of cultural and language barriers on participant recruitment.
2. Incorporate a long‐term follow‐up plan: conduct follow‐ups with patients at 6 months and 12 months after they complete the programme, respectively, to collect data on patients' health status, quality of life, adherence to exercise and other relevant indicators.
3. Develop a more detailed and comprehensive assessment questionnaire that covers lifestyle dimensions, including dietary structure and daily exercise habits. During the data analysis phase, these factors should be incorporated into the statistical model as confounding variables to more accurately determine the relative merits of the two pulmonary rehabilitation models themselves and enhance the credibility of the conclusions.

In fact, our team has also conducted several clinical trials on the effectiveness of pulmonary rehabilitation for COPD patients. However, due to various reasons, the rate of patient loss to follow‐up remains persistently high, which has caused significant inconveniences to both our clinical management and research efforts. Therefore, we are very pleased to see this study focusing on patients' psychological experiences, as it provides a new perspective for us to address the issue of lost follow‐ups.

I appreciate the opportunity to share my thoughts and commend Hannah Rutherford et al. for their impactful contribution. I hope these suggestions will inspire further research to advance understanding in this field. I also look forward to conducting in‐depth exchanges with Hannah Rutherford's team on topics such as ‘mHealth adaptation solutions for populations with low digital literacy’ and ‘practical strategies for long‐term follow‐up’, so as to jointly promote research progress in the field of pulmonary rehabilitation for COPD. Additionally, I hope to have the opportunity to engage in in‐depth discussions with all fellow researchers in this area.

## Author Contributions

**Wen Sun:** methodology, writing – original draft, writing – review and editing. **Caixia Qiu:** methodology, writing – review and editing. **Shungui Xu:** supervision, funding acquisition, project administration, writing – review and editing.

## Conflicts of Interest

The authors declare no conflicts of interest.

## Data Availability

The authors have nothing to report.
